# Recurrent Thrombotic Thrombocytopenic Purpura-Like Syndrome as a Paraneoplastic Phenomenon in Malignant Peritoneal Mesothelioma: A Case Report and Review of the Literature

**DOI:** 10.1155/2012/619348

**Published:** 2012-10-02

**Authors:** Francisco Socola, Arturo Loaiza-Bonilla, Ernesto Bustinza-Linares, Ricardo Correa, Joseph D. Rosenblatt

**Affiliations:** Division of Hematology, Department of Medicine, Jackson Memorial Hospital/Sylvester Comprehensive Cancer Center, University of Miami Miller School of Medicine, 1475 NW 12th Avenue Suite 3300, Miami, FL 33136, USA

## Abstract

We report the case of an African American male with no significant past medical history presenting with recurrent, rapidly relapsing episodes of thrombotic thrombocytopenic purpura (TTP) despite aggressive treatment with several lines of treatment. Incidentally, these episodes were associated with severe abdominal pain which eventually developed into acute abdomen and prompted exploratory laparotomy, revealing diffuse carcinomatosis with a tumor located on the left pelvis that was encasing the distal sigmoid colon. Pathology made a final diagnosis of peritoneal mesothelioma. TTP-like syndrome (TTP-LS) has been described as a paraneoplastic phenomenon in several malignancies but never before in the setting of malignant mesothelioma. Paraneoplastic TTP-like syndrome has historically been associated with a dismal prognosis and particular clinical and laboratory abnormalities described in this paper. It is of utmost importance to make a prompt determination whether TTP is idiopathic or secondary to an underlying condition because of significant differences in their prognosis, treatment, and response. This paper also reviews the current literature regarding this challenging condition.

## 1. Case Presentation

A 61-year-old African American male with no significant past medical history presented to our institution's emergency department complaining of six-month history of progressively worse abdominal pain associated with anorexia and unintended weight loss. Computerized tomography (CT) scan of abdomen showed mild ascites and right pleural effusion; a subsequent CT of the chest revealed a large right pleural effusion with multiple subcentimeter (less than 4 millimeters) pulmonary nodules in the right upper and lower lobes. Initial laboratory workup revealed a complete blood count (CBC) with normochromic normocytic anemia (hemoglobin 7.3 g/dL), thrombocytopenia (platelets 36,000), and reticulocytosis (10%). Lactate dehydrogenase (LDH) was found elevated with a low haptoglobin. Coombs test was negative, and renal and liver functions were within normal limits. A peripheral blood smear confirmed CBC findings and showed schistocytes and microspherocytes. Given the likely diagnosis of thrombotic thrombocytopenic purpura (TTP), ADAMTS 13 activity was ordered and reported as 14% (low), which led to the initial confirmation of this disorder. The patient was started on high-dose steroids, plasmapheresis (PE), and fresh frozen plasma (FFP). After seven days of treatment, his platelet count increased to 428,000 and LDH decreased to 839. Extensive workup for secondary causes of TTP including malignancy was negative. Human immunodeficiency virus and hepatitis serologies were nonreactive. Pleural fluid analysis of right pleural effusion was consistent with an exudate; however, cytology and flow cytometry were negative for malignant cells. Quantiferon for tuberculosis was positive but acid fast bacilli stains and viral, fungal, mycobacterial, and bacterial cultures were negative. The possibility of latent tuberculosis was raised, and the patient was started on isoniazid. Once the patient was stable, video-assisted thoracoscopic surgery (VATS) with multiple pleural biopsies was done; however, no lesions were seen during the procedure, and pathology did not reveal any evidence of malignancy. Plasmapheresis and FFP were discontinued, steroids were tapered off, and eventually the patient was discharged with outpatient followup.

After 9 days, the patient returned to the emergency department complaining of worsening of abdominal pain and altered mental statues with dysarthria and bradypsychia. Once again, he was found thrombocytopenic (<10,000) and his LDH was very high (4000). CT brain showed no hemorrhage and electroencephalogram (EEG) revealed encephalopathy but no epileptogenic foci were found. The patient was admitted to medical intensive care unit, and PE and FFP infusions were started after a new peripheral blood smear led to confirmation of relapsed TTP. Subsequent magnetic resonance imaging (MRI) of the brain did not show any abnormalities. The patient eventually recovered with instaurated treatment, and he was discharged again with oral steroids. ADAMTS 13 activity at the time of discharge was 19%. However, after only 6 days, he was admitted again complaining of the same symptoms of abdominal pain and altered mental status. He was also found febrile, oliguric, and azotemic. Relapsed TTP was once again diagnosed, and ADAMTS13 activity was reported to be less than 10%. During his hospital stay he had two episodes of seizures and for that reason he was started on levetiracetam. EEG showed diffuse encephalopathy. Following seven days of treatment with PE and FFP, the patient had a significant overall improvement, and, after no hemolysis was found, the patient was transitioned to daily FFP infusions. Despite this, ADAMTS activity remained low, and, once FFP infusions were discontinued, his TTP relapsed again. After reinitiating treatment, rituximab 375 mg/m^2^ was started as second-line therapy.

**Figure 1:**
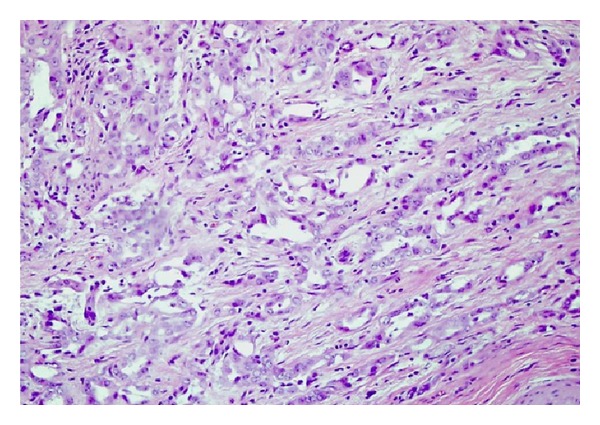
Abdominal mass biopsy showing mesothelioma (H&E, low power).

**Figure 2:**
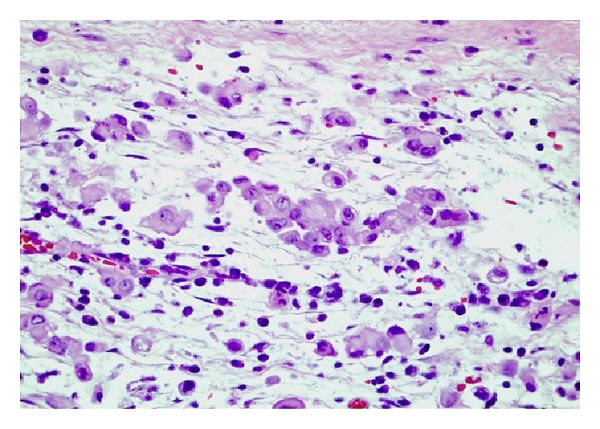
Mesothelioma (H&E, high power).

**Figure 3:**
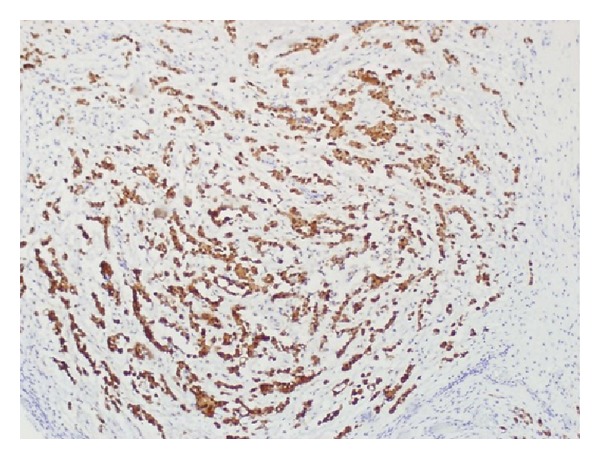
Immunohistochemistry showing calretinin-positive cells.

**Figure 4:**
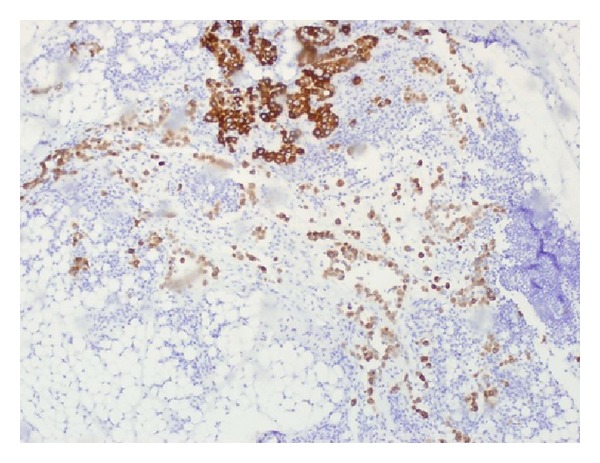
Immunohistochemistry showing cytokeratin-positive cells.

**Figure 5:**
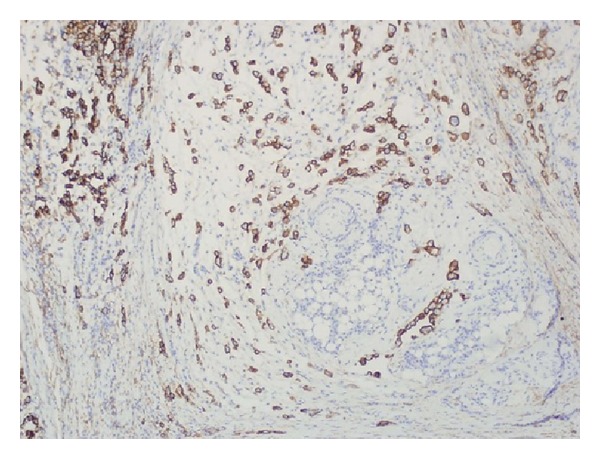
Immunohistochemistry showing D240-positive cells.

After seven days of immunotherapy along with PR and FFP, ADAMTS13 remained persistently low (10%). Chemotherapy with CVP (cyclophosphamide, vincristine, and prednisone) was added for empiric treatment of potential low-grade lymphoma, with no significant response after two cycles of immunochemotherapy. He started complaining again of intense abdominal pain leading to acute abdomen that prompted an exploratory laparotomy. Surgical exploration revealed diffuse carcinomatosis with a tumor located on the left pelvis that was encasing the distal sigmoid colon. Pathology reported mesothelioma with malignant cells positive by immunohistochemistry to calretinin and D240, and negative for CEA and MOC31 (Figures 1, 2, 3, 4, and 5). Due to the extension of his disease, it was considered nonresectable. Based on his poor response to TTP treatment and concomitant primary malignancy, the diagnosis of paraneoplastic TTP-like syndrome (TTP-LS) was made. The patient continued with FFP while recovering from surgery, and then he was started on palliative chemotherapy with cisplatin plus pemetrexed, the best chemotherapy available for mesothelioma, aiming to control both his TTP-LS and malignancy. After four weeks of treatment, there were no signs of improvement and he was transferred to hospice to receive best supportive care.

## 2. Discussion

Thrombotic thrombocytopenic purpura (TTP) is a life-threatening disseminated thrombotic microangiopathy, occurring mainly in adults and characterized by thrombocytopenia, hemolytic anemia, neurologic disturbances, renal abnormalities, and fever [1]. The incidence for idiopathic TTP is estimated to be approximately 3.7 annually per million in the USA and it is usually more prevalent in women and young people [1]. The most common laboratory findings are the presence of predominantly intravascular microangiopathic hemolytic anemia (peripheral blood smear showing schistocytes—helmet cells), elevated LDH secondary to hemolysis and systemic ischemia, reticulocytosis, presence of nucleated red blood cells on peripheral smear, unconjugated hyperbilirubinemia, low plasma haptoglobin, thrombocytopenia (often severe < 20 × 10^9^/L), negative Coombs test, and normal coagulation tests [2–5].

Typically the pathogenesis of idiopathic ITP is associated with decreased activity or deficiency of ADAMTS-13 (Von Willebrand factor-cleaving protease) secondary to an IgG inhibitory autoantibody or rarely to a congenital deficiency. This decreased protein activity produces accumulation of “unusually large” Von Willebrand factor (VWF) multimers leading to platelet aggregation and microthrombi formation of agglutinated platelets, occluding arterioles, and capillaries [2–5]. If untreated, TTP carries a mortality rate above 90% secondary to life-threatening complications such as seizures, confusion, transient ischemic attack [2], chronic renal failure (especially in TTP-hemolytic uremic syndrome) [6], intestinal ischemia, retinal detachment [7], myocardial infarction [8], multiorganic dysfunction, sepsis, shock, and thrombosis. Due to its significant morbidity and mortality, solely the presence of microangiopathic hemolytic anemia and thrombocytopenia without any other apparent cause is considered sufficient for making the diagnosis of TTP and prompts early treatment with plasmapheresis/plasma exchange. Plasma exchange works by removing autoantibodies to ADAMTS-13 when present, depleting the circulating very high molecular VWF multimers, and replenishing the missing protease (ADAMTS-13) in the plasma [9]. Treatment can reduce the mortality rate to approximately 20% [10].

Although many cases are idiopathic, TTP may be secondary to cancer, drugs as quinine [3], ticlopidine [11], clopidogrel [12], chemotherapeutic agents as mitomycin C, gemcitabine, immunosuppressant agents cyclosporine A, tacrolimus [13], valacyclovir [14], congenital ADAMTS-13 deficiency, acute inflammatory states such as pancreatitis or HIV infection [3], and it has also been described in pregnancy and allogeneic hematopoietic stem cell transplant patients [15]. It is very important to determine whether TTP is idiopathic or secondary to an underlying condition because there are differences in their prognosis, treatment, and response.

The initial diagnosis of TTP may be uncertain because several other disorders can produce similar symptoms and laboratory findings. In idiopathic TTP, ADAMTS-13 activity can be severely deficient (<5%), but Francis et al. reported that 70% of 133 patients with idiopathic TTP did not have severely deficient ADAMTS-13 activities. Furthermore, the same authors reported that patients suffering from disseminated malignancy may have lower ADAMTS-13 activities than normal due to high plasma levels of Von Willebrand factor [16]. Malignancy as the cause for symptoms leading to the diagnosis of TTP has been reported but was often diagnosed only after autopsy. The presence of a primary malignancy is an important consideration in the differential diagnosis of TTP, particularly in patients with refractoriness to first-line therapy with PE and FFP infusions; however, sometimes the malignancy is not clinically apparent (e.g., our case), leading to an initial diagnosis of idiopathic TTP and subsequent PE treatment [16]. Furthermore, failure to urgently initiate plasma exchange treatment in a patient with idiopathic TTP may result in significant morbidity and mortality, which justifies and warrants such treatment pending further investigation [16]. Nevertheless, leaving a disseminated malignancy undiagnosed exposes the patient to the major risks of plasma exchange (central venous catheter insertion-related sepsis, thrombosis, bleeding, and pericardial tamponade) and may delay potentially curative and/or life-prolonging chemotherapy [17].

Secondary TTP and TTP-like syndromes despite their rarity have been described as paraneoplastic phenomena and primary manifestation in several malignancies. In the Oklahoma TTP-HUS Registry, 10 patients (3% of TTP patients) were initially diagnosed with idiopathic TTP and subsequently systemic malignancy was discovered. The median age of these patients was 56 years; seven patients were men, one patient had history of breast cancer, but she was thought to be cancer-free at the time of presentation. The most common presenting symptoms were weakness, dyspnea, cough, fever, and abdominal pain. The median duration of symptoms prior to the initial diagnosis of TTP in all 10 patients was 21 days (range, 1–85 days); two had severe neurologic abnormalities and six had confusion, three of them had acute renal failure that required dialysis. All patients had increased LDH levels; four had values exceeding 5,000 U/L. Coagulation profile was normal in 7 patients. ADAMTS-13 activity was measured in eight patients; the median value was 50% (range, 13%–100%). The median number of plasma exchange treatments was five (range, 1–9), and only one responded to PE. Disseminated malignancy was diagnosed 2–14 days (median, 6 days) after the initial diagnosis of TTP, and it was determined by bone marrow biopsy in six patients [16].

Regarding their prognosis, all 10 patients died soon after the initial diagnosis of TTP (median, 12 days; range, 3–75 days). Of the eight patients who were diagnosed before death, only one received chemotherapy, but he died 6 days after starting treatment, 4 patients died before chemotherapy, and 3 were too ill to receive it. The final diagnoses were breast carcinoma, non-small-cell lung cancer, pancreatic carcinoma, renal carcinoma, myelodysplasia (refractory anemia with excess blasts), acute lymphoblastic leukemia, diffuse large B-cell lymphoma, and Kaposi's sarcoma [16].

In the same registry, when compared idiopathic versus paraneoplastic TTP-LS, the latter group of patients were predominantly males, they had a longer duration of symptoms before the diagnosis of TTP, increased frequency of respiratory symptoms at presentation (dyspnea and cough), and greater elevations of serum LDH. These patients also had a poorer response to treatment and a significantly higher mortality rate. No significant differences were found in age, race, frequency of neurologic or renal abnormalities, severity of anemia and thrombocytopenia, or ADAMTS-13 activity (no patients had a severely deficient ADAMTS-13 activity ≤ 5%) [16].

In reported cases throughout the English literature, 19 patients were identified with TTP-like syndromes associated to underlying malignancy, 11 were males; the median age was 58 years, and 14 patients had no previous diagnosis of cancer. The median duration of symptoms, reported for 11 patients, was 21 days. Six patients had presenting symptoms of pain other than abdominal (four back pain, one bone pain, and one jaw pain). Four patients had severe neurologic abnormalities. The median time from the initial presentation to the diagnosis of malignancy was 14 days. In six patients, the malignancy was diagnosed by bone marrow biopsy. The primary cancers were identified only in 16 of these 19 patients and were gastric carcinoma, prostate carcinoma, carcinoma of unknown primary, anal squamous cell carcinoma, and colon carcinoma, and multiple endocrine neoplasia type I. It is important to note that 9 out of 19 previously reported patients received chemotherapy and five achieved remission, with most of these malignancies being mucin-rich adenocarcinomas [18–35]. The latter finding suggests that treatment of the underlying malignancy may be the best therapeutic option in paraneoplastic TTP-LS given its poor response to standard treatment aiming to replenish ADAMTS 13 levels. Further studies to confirm this hypothesis are needed; however, the rarity and underreporting of such cases makes a prospective study a difficult task.

## 3. Conclusion

In this paper, we report the case of a male with relapsing, refractory paraneoplastic TTP-like syndrome. To the best of our knowledge, this is the first reported case of such phenomenon secondary to peritoneal mesothelioma. Our case has several particular characteristics including a difficult-to-diagnose underlying malignancy, the rarity of the tumor, and the poor response of both malignancy and paraneoplastic TTP-LS to several lines of treatment including high dose chemotherapy. The persistence of low ADAMTS-13 activity despite clinical and hematological recovery suggests a different pathogenesis and clinical course compared to the autoimmune theory commonly attributed to primary idiopathic TTP. Thrombotic thrombocytopenic purpura-like syndrome has been described as a paraneoplastic phenomenon in several malignancies, and it has been historically associated with a dismal prognosis and particular clinical and laboratory abnormalities as described above. It is of utmost importance to make a prompt determination whether if TTP is idiopathic or secondary to an underlying condition because of differences in their prognosis, treatment, and response. Having a prompt diagnosis of paraneoplastic TTP-LS provides an opportunity for treatment with appropriate early and potentially curative or life-prolonging chemotherapy and to avoid unnecessary interventions or potential complications. The infrequency with which paraneoplastic TTP-like syndrome is encountered and its pathophysiology make it a formidable diagnostic and therapeutic challenge.
